# Species-specific effects of understory shrubs on soil chemistry and microbial communities in temperate Scots pine forests

**DOI:** 10.1186/s12870-026-09514-6

**Published:** 2026-07-14

**Authors:** Jarosław Lasota, Marta Kempf, Ewa Błońska

**Affiliations:** Department of Ecology and Silviculture, Faculty of Forestry, University of Agriculture, 29 Listopada 46 Str., Krakow, 31-425 Poland

**Keywords:** Bacteria, Fungi, Forest soil, Shrubs, Soil microorganisms, NGS

## Abstract

**Background:**

Understory shrubs are key components of forest ecosystems that influence belowground processes by modifying soil properties and shaping microbial communities through litter input and root exudation, yet their role in regulating soil microbial diversity remains less understood than that of trees. This study investigated how understory shrubs affect soil chemistry and the diversity and composition of bacterial and fungal communities in temperate pine (*Pinus sylvestris*) forests across organic and mineral soil horizons. Soil samples were collected from pine stands with dominant shrubs—rowan (*Sorbus aucuparia*), alder buckthorn (*Frangula alnus*), and European hazelnut (*Corylus avellana*)—and analysed using chemical assays and next-generation sequencing. A total of 20 study plots were included in the experiment, with each stand variant represented by five independent plots.

**Results:**

Soils under shrubs exhibited higher pH levels, greater nutrient availability, and significantly higher microbial diversity compared to pine monocultures. The presence of understory shrubs was associated with higher fungal and bacterial taxonomic richness compared to pure pine stands, with the strongest effect observed in stands with European hazelnut understory. The dominant fungal phyla were Ascomycota, Basidiomycota, and Mortierellomycota, while bacterial communities were mainly composed of Actinobacteriota, Proteobacteria, and Acidobacteriota. Principal component analysis indicated that soil pH and concentrations of carbon, nitrogen, calcium, and magnesium were key factors shaping microbial community composition. The most distinct and diverse microbial assemblages were observed in soils under European hazelnut.

**Conclusion:**

These findings demonstrate that shrubs enhance soil fertility and microbial diversity by improving nutrient cycling and microhabitat conditions. Integrating shrub species, particularly European hazelnut, into coniferous stands may strengthen belowground biodiversity and enhance the stability and long-term functioning of forest ecosystems.

**Supplementary Information:**

The online version contains supplementary material available at https://doi.org/10.1186/s12870-026-09514-6.

## Background

 Forest biodiversity encompasses not only trees but also a diverse range of organisms, including understory plants, shrubs, animals, and, in particular, soil microorganisms [1]. This complex web of life underpins forest ecosystem functioning and resilience. Among these components, soil microorganisms play a pivotal role in driving biogeochemical cycles, decomposing organic matter, and facilitating the transformations of essential nutrients for plant growth. Soil is one of the largest reservoirs of biodiversity on Earth, with one gram of forest soil containing billions of bacterial cells and thousands of microbial taxa, particularly bacteria and fungi, as well as numerous soil fauna organisms such as nematodes and arthropods, all of which contribute to ecosystem functioning and stability [2, 3].

However, climate change, driven by shifts in temperature regimes, altered precipitation patterns, and increased extreme weather events, exerts growing pressure on forest ecosystems and the microorganisms inhabiting them [4, 5]. These abiotic stressors affect microbial activity and diversity both directly and indirectly, influencing decomposition rates, soil respiration, and nutrient availability [6]. The stability and resilience of microbial communities are thus crucial indicators of forest health [7]. Biodiverse forest stands, which contain a variety of plant functional types, including shrubs, tend to be more productive, more resilient to disturbance, and better adapted to changing environmental conditions [8].

Among forest understory components, shrubs are a key group that influences belowground ecological processes [9]. By contributing aboveground litter and belowground root exudates, shrubs actively modify soil physicochemical properties, affect microbial biomass, and shape microbial community structure [10]. Shrub litter serves as a substantial source of organic matter, enhancing the availability of carbon and nutrients to soil microbes and promoting enzymatic activity involved in decomposition and mineral cycling [11, 12]. The chemical composition of shrub litter, including its carbon-to-nitrogen ratio, lignin content, and secondary metabolites, varies significantly across species and determines the rate and pathway of decomposition [13]. These differences directly affect microbial colonisation patterns, enzyme production, and the availability of mineral nutrients [14–16]. For instance, litter rich in phenolic compounds may inhibit certain decomposers, while nitrogen-rich inputs can stimulate microbial activity. Decomposition of litter is central to carbon and nutrient cycling, contributing to humus formation and influencing long-term soil fertility. In addition to litter-derived inputs, shrubs may influence soil microbial communities through belowground processes, including fine-root turnover and the release of root-derived organic compounds into the surrounding soil [10]. These inputs provide readily available carbon sources, such as sugars, amino acids, and organic acids, which can stimulate microbial activity and short-term soil biochemical processes [17, 18]. Root-derived organic inputs may also contribute to the formation and stabilisation of soil organic matter (SOM) through the accumulation of microbial residues involved in long-term carbon storage [19, 20]. As a result, soils under shrub understories may create favourable conditions for microbial biomass development and significantly influence microbial community composition and enzymatic activity [21]. In addition to direct organic inputs, shrubs indirectly influence soil microbial communities by modifying microclimatic conditions (e.g., shading, soil moisture), altering pH, and altering nutrient profiles, including total C, N, and P content. They can also affect the accumulation of allelopathic compounds such as phenolic acids, which may inhibit or stimulate specific microbial groups [22]. These interactions highlight that shrubs are not merely passive structural elements, but active ecological agents that shape the soil environment and support belowground biodiversity and biogeochemical processes. Since most fine roots and freshly decomposing organic material are concentrated in the upper soil horizons, microbial communities inhabiting these horizons are generally considered more responsive to plant-driven changes in nutrient availability and substrate quality [23, 24].

Although the ecological importance of shrubs in forest ecosystems has increasingly been recognised, their influence on belowground processes and soil microbial communities remains less extensively studied than that of canopy tree species. The novelty of this study lies in the simultaneous analysis of two soil horizons and the comparison of the effects of three understory shrub species on soil physicochemical properties and bacterial and fungal communities. Therefore, the novelty of the present study lies in (i) comparing the effects of three ecologically distinct shrub species (European hazelnut, alder buckthorn, and rowan) on soil microbial communities, (ii) simultaneously assessing responses in both organic and mineral soil horizons, and (iii) relating microbial community composition and diversity to accompanying changes in soil chemical characteristics. This integrated approach provides new insight into how different understory shrub species are associated with belowground biological and chemical processes in temperate pine forests. Shrub establishment increased the spatial variability of soil resources, such as water and nutrient availability, thereby influencing plant community composition and functioning [25]. Using DNA metabarcoding and analyses of plant traits, soil properties, and enzyme activity, the study demonstrated that shrub encroachment significantly alters soil fungal community composition and functional structure, with these changes being strongly linked to shrub density, plant tissue quality, and soil chemical conditions [26]. To date, research on shrub effects has largely concentrated on a limited number of species and narrowly defined soil characteristics, with comparatively little attention given to their integrated influence on soil chemistry, microbial diversity, and ecosystem functioning. Therefore, it is important to investigate their role in shaping soil microbial communities and driving biogeochemical cycles. A better understanding of these interactions may provide valuable insights for biodiversity-oriented forest management and climate adaptation strategies. The primary objective of this study is to investigate the influence of shrubs on the diversity and structure of soil microbial communities in temperate pine forests. In the conducted study, we hypothesise that: H1: the presence of shrubs in the temperate pine forest alters the microbial composition of the soil; H2: soil properties shaped by shrubs, such as pH and nutrient content, are among the key factors determining the composition and diversity of soil bacterial and fungal communities; H3: the responses of soil microbial abundance in the organic horizon of soil were more sensitive to shrubs’ influence than those in deeper soil horizons.

## Methods

### Study sites and soil sampling

This study was conducted in five forest districts: Olkusz, Koniecpol, Włoszczowa, Przedbórz, and Smardzewice in Poland, with formal permission granted by the respective forest districts acting as land managers of the study sites. Geographic information, including longitude and latitude, was recorded for each collection site (Table S1). Study plots were selected to ensure the highest possible homogeneity of habitat and stand conditions. All plots were located within the same forest site type and were characterised by similar soil origin and texture, stand age, tree density, canopy closure, and topographic conditions. Plot selection was intended to minimise the influence of environmental gradients unrelated to understory shrub composition, such as differences in soil moisture, microrelief, or light availability. Research plots measuring 0.05 ha were established on fluvioglacial sandy soils, predominantly classified as Podzols, with acidic ectohumus. All plots shared similar soil texture, with an average composition of sand (90 ± 9%), silt (7 ± 4%), and clay (3 ± 2%). The investigation included four stand variants: pure Scots pine stands without y shrubs and Scots pine stands with understory layers dominated by rowan (*Sorbus aucuparia*), alder buckthorn (*Frangula alnus*), or European hazelnut (*Corylus avellana*). Variant with shrubs consisted of pine with a single dominant shrub species. All stands were of similar age (80 years) and had moderate canopy density (narrow crown gaps). Age was confirmed via forest management maps. Dense shrub biogroups formed the understory, each covering 0.05 ha, with shrub species comprising approximately 90% of each plot. The study was conducted at five locations (Table S1). At each location, four plots were established, representing the following understory variants: pure Scots pine stands without shrubs, and Scots pine stands with understory layers dominated by rowan, alder buckthorn, or European hazelnut. Consequently, the experimental design comprised 20 plots (5 locations × 4 variants of study plots).Soil sampling was conducted during the second half of June 2024. For each study plot, ten subsamples were collected and combined into a single composite sample representative of the analysed shrub species and soil horizon. Soil samples were collected from two horizons (organic O and humus mineral horizon A). The O horizon was identified in the field as the surface organic horizon composed predominantly of undecomposed and partially decomposed plant material, characterised by a high organic matter content (> 20–30%). The A horizon was defined as the underlying humus mineral horizon, onsisting mainly of mineral material enriched with humified organic matter. Horizon differentiation was based on visible changes in colour, structure, organic matter content, and the transition from organic to mineral material rather than on fixed depth criteria. Samples were placed in plastic bags, labelled, and tools were disinfected with ethanol (> 99%) before each sampling. Samples were transported to the lab and stored in the dark at 4 °C. All samples were sieved to a 2 mm mesh size before analysis.

### Chemical characteristics

All soil chemical analyses were performed on air-dried soil samples that had been homogenised and passed through a 2-mm sieve prior to analysis. Soil pH was measured potentiometrically in a soil-to-water suspension at a 1:2.5 (w/v) ratio. The suspension was allowed to equilibrate for 24 h before pH was measured potentiometrically. Total organic carbon (C) and total nitrogen (N) contents were determined using a LECO CNS TrueMac Analyser (Leco, MI, USA). Exchangeable base cations (Ca²⁺, Mg²⁺, K⁺, and Na⁺) were extracted from soil samples using 1 M ammonium acetate solution (pH 7.0) and quantified by inductively coupled plasma optical emission spectrometry (ICP-OES; Thermo iCAP 6500 DUO, Thermo Fisher Scientific, USA). Prior to analysis, soil extracts were filtered to remove suspended particles.

### Fungal and bacterial DNA library preparation, sequencing, and bioinformatic analysis

The soil mycobiome samples that were collected for analysis were stored at − 80 °C. DNA was isolated from 1 g of soil using the Genomic Mini AX Bacteria + kit (A&A Biotechnology, Poland) according to the manufacturer’s protocol. Mechanical lysis was performed using zirconia beads in a FastPrep-24 homogeniser. Additionally, enzymatic lysis was carried out using lyticase (A&A Biotechnology, Poland). Fungal DNA libraries were prepared by amplifying the ITS1 rDNA region with primers ITS1F [27] and ITS2 [28], following the “16S Metagenomic Library Preparation” protocol (Illumina). Bacterial DNA libraries were generated by amplifying the V3–V4 region of the 16 S rRNA gene using primers 341 F and 785R [29]. PCR was conducted in a reaction mixture containing 15 ng of genomic DNA using the Q5 Hot Start High-Fidelity 2X Master Mix (New England Biolabs, USA). Indexing PCR was performed using the Nextera XT index kit (Illumina). Following indexing, the libraries were purified using AMPure XP beads, and their quality and concentration were assessed using a Bioanalyzer (Agilent Technologies, USA) and quantitative PCR (qPCR). Sequencing of DNA libraries was performed on the Illumina MiSeq platform (2 × 300 bp, paired-end) by Genomed (Poland), with a sequencing depth of 50,000 reads per sample.

NGS data for fungi were analysed using QIIME v1.9.1 [30]. Samples were demultiplexed, and fastq files were generated using MiSeq Reporter v2.6 (Illumina). Adapter and low-quality sequences (below Q20) were removed using cutadapt [31]. Paired reads were merged using the seqprep algorithm. Chimeric sequences were removed using Usearch61 [32]. Fungal reads were clustered using the uclust algorithm [32] and matched against the UNITE v8.2 ITS database (UNITE Community, 2018) using the BLAST algorithm [33]. Operational taxonomic units (OTUs) with very low abundance were filtered out; only those with a relative abundance of at least 0.01% were included in further analyses. Fungal sequences were clustered into OTUs at 97% similarity using the uclust algorithm. Based on the occurrence of fungal species, their relative abundances were calculated, and a heatmap (with Euclidean cluster analysis) was generated using log10 + 1 transformed abundance data for fungal species with more than 500 reads in at least five plots of a given shrub species.

NGS data for bacteria were also processed using QIIME v1.9.1 [30]. Samples were demultiplexed, and fastq files were generated using MiSeq Reporter v2.6 (Illumina). Adapter and low-quality sequences were removed with cutadapt [31]. Paired reads were merged and clustered using the DADA2 v1.18 algorithm [34], which was also used for chimera removal. The resulting amplicon sequence variants (ASVs) were matched against the 16 S Silva 138 database [35]. Further processing and analysis of bacterial ASVs followed the same approach as for fungal OTUs.

Libraries with the fungal sequences analysed have been deposited with the GenBank (https://www.ncbi.nlm.nih.gov/) under project number PRJNA1357095, and those with the bacterial sequences have been deposited under project number PRJNA1357109.

### Statistics

Statistical analyses were performed using R (R Core Team, 2020) and RStudio. Spearman correlations were calculated between the total number of microorganisms (OUT) and soil properties. PCA was used to explore relationships between soil chemistry and microorganisms. Normality was tested using the Shapiro-Wilk test; homogeneity of variance was assessed with Levene’s test. To assess the influence of species and soil horizons on microorganism taxonomic richness, a generalised linear model (GLM) was used. The significance threshold was set at *p* < 0.05. Data visualisation was performed using the readxl, dplyr, scales, ggplot2, and pheatmap packages. Spearman correlation analyses were employed with corrplot, while PCA was conducted with the MASS, factoextra, and ggfortify packages. Differences in microorganism community composition among shrub species were analysed using permutational multivariate analysis of variance (PERMANOVA) based on Bray–Curtis dissimilarity matrices, implemented with the adonis2 function in the vegan package. Prior to PERMANOVA, homogeneity of multivariate dispersion was evaluated using the betadisper function. For each pairwise comparison, pseudo-F statistics, the proportion of explained variance (R²), and permutation-based p-values were calculated. Statistical significance was accepted at *p* < 0.05. Non-metric multidimensional scaling (NMDS) based on Bray–Curtis distances was additionally performed to visualise patterns of fungal community similarity among samples. Hypothesis H1, assuming that the presence of shrubs alters soil microbial community composition, was tested using PERMANOVA and NMDS analyses based on Bray–Curtis dissimilarity matrices. Hypothesis H2, concerning the influence of shrub-mediated soil properties on microbial diversity and composition, was evaluated using Spearman correlation analyses and PCA ordination relating microbial taxa to soil chemical parameters. Hypothesis H3, stating that microbial responses in organic horizons are more sensitive to shrub influence than those in mineral horizons, was assessed using generalised linear models (GLMs), with shrub species, soil horizon, and their interaction included as explanatory variables. Alpha diversity of fungal and bacterial communities was evaluated using the Shannon diversity index (H), Simpson diversity index (D), and Pielou’s evenness index (J) [36]. The Shannon index was used to assess overall community diversity by accounting for both taxon richness and relative abundance, whereas the Simpson index reflected the dominance structure of microbial communities. Pielou’s evenness index was calculated to estimate the uniformity of taxon distribution within individual samples. All indices were calculated from relative abundance data obtained from NGS sequencing. Fungal functional guilds were assigned using the FUNGuild database (v1.1) based on genus-level taxonomic assignments, as species-level identification was not consistently available across all taxa [37]. Only taxa with “Probable” or “Highly Probable” confidence rankings were included in downstream functional analyses to reduce uncertainty in functional classification. Relative abundances of functional guilds were calculated per sample by summing taxon abundances and expressing them as percentages of the total fungal community. For visualisation and comparative analyses, functional assignments were summarised into major trophic modes (saprotrophs, symbiotrophs, pathotrophs and others).

## Results

### Effects of shrub species on soil chemical properties

Nitrogen and carbon contents were highest in the organic (O) horizons and lowest in the humus-mineral (A) horizons. The highest concentrations of both N (1.75%) and C (36.09%) were recorded under alder buckthorn, whereas the lowest were found under pine (0.12% N and 1.82% C) (Table 1). Soil pH ranged from 3.37 under rowan to 4.77 under European hazelnut. Calcium concentrations were highest under European hazelnut, while potassium and magnesium were highest under alder buckthorn. Overall, soils under alder buckthorn and European hazelnut exhibited the most favourable chemical properties, whereas pine soils were the least fertile and most acidic (Table 1).

**Table 1:** Chemical properties of the tested soils depending on the species

Species	Horizon	*N*	C	pH	Ca	K	Mg	Na
AB	O	1.75 ± 0.26	36.09 ± 7.27	3.76 ± 0.17	11.16 ± 2.18	0.76 ± 0.12	1.54 ± 0.38	0.22 ± 0.03
	A	0.24 ± 0.02	3.26 ± 0.48	3.53 ± 0.04	0.62 ± 0.09	0.07 ± 0.02	0.1 ± 0.03	0.06 ± 0.0
EH	O	1.52 ± 0.24	28.77 ± 6.7	4.47 ± 0.13	25.22 ± 4.95	0.81 ± 0.24	2.79 ± 0.79	0.09 ± 0.02
	A	0.43 ± 0.09	8.39 ± 2.33	3.94 ± 0.06	4.01 ± 0.49	0.16 ± 0.07	0.47 ± 0.09	0.03 ± 0.0
RW	O	1.06 ± 0.11	17.11 ± 3.36	3.95 ± 0.13	6.50 ± 1.62	0.59 ± 0.16	0.74 ± 0.17	0.07 ± 0.01
	A	0.28 ± 0.03	4.17 ± 1.01	3.73 ± 0.17	0.93 ± 0.2	0.09 ± 0.02	0.12 ± 0.02	0.02 ± 0.0
PN	O	0.97 ± 0.21	33.51 ± 8.28	3.57 ± 0.05	4.52 ± 0.64	0.24 ± 0.03	0.02 ± 0.0	0.05 ± 0.0
	A	0.12 ± 0.02	1.82 ± 0.53	3.76 ± 0.13	0.11 ± 0.02	0.02 ± 0.01	0.0 ± 0.0	0.02 ± 0.0

mean ± SD; AB – alder buckthorn, EH – European hazelnut, RW – rowan, PN - pine; C – carbon content [%]; N – nitrogen content [%]; exchangeable cation content Ca, K, Mg, Na – [cmol(+).kg-1]; O – organic horizon, A – humus mineral horizon

The obtained statistical values indicate that both species and soil horizon had a statistically significant effect (*p* < 0.001) on all analysed chemical properties (Table 2). The GLM analysis revealed a statistically significant interaction between shrub species and soil horizon (S × H) for all analysed parameters (*p* < 0.001; Table 2).

**Table 2:** Summary of the GLM analysis for the examined properties in the soil under the influence of various species

	*N*		C		pH		Ca		K		Mg		Na	
	F	*p*	F	*p*	F	*p*	F	*p*	F	*p*	F	*p*	F	*p*
Species (S)	19.74	0.0000	6.85	0.0000	44.81	0.0000	72.54	0.0000	17.79	0.0000	45.92	0.0000	169.02	0.0000
Horizon (H)	445.51	0.0000	243.91	0.0000	25.98	0.0000	251.43	0.0000	197.77	0.0000	113.75	0.0000	391.50	0.0000
S*H	10.51	0.0000	9.23	0.0000	14.61	0.0000	34.96	0.0000	8.92	0.0000	24.20	0.0000	59.28	0.0000

A statistically significant effect is marked in red

### Effects of shrub understory on the taxonomic and functional structure of soil microbial communities

**Fig. 1:**
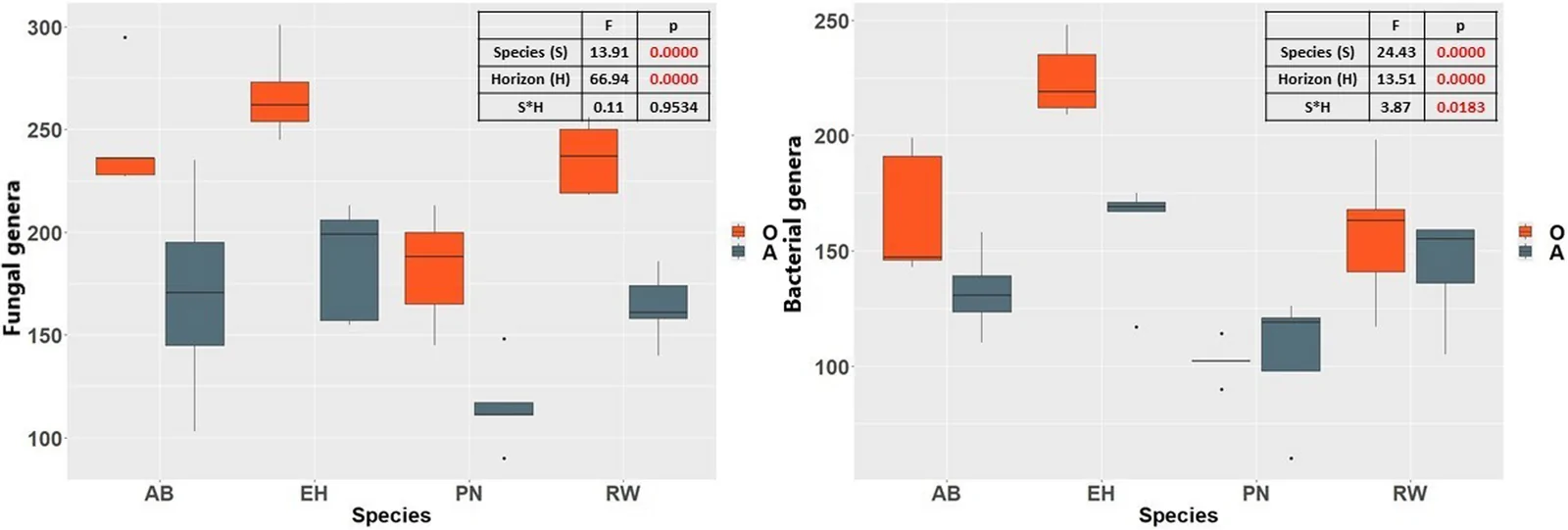
Total number of fungal and bacterial taxa identified in soil under influence of different species (AB – alder buckthorn, EH – European hazelnut, RW – rowan, PN – pine; O – organic horizon, A – humus mineral horizon)

The total number of fungal and bacterial genera identified by NGS also varied between the examined stand variants (Fig. 1). Regardless of shrub species, the organic horizons (O) were characterised by a higher number of detected fungal genera compared to the humus mineral horizons (A). Only in pine monocultures was the number of bacterial genera similar between the two genetic horizons. In the soils of pine monocultures (without the presence of shrubs), the lowest number of fungal genera was recorded, on average 182 in the O horizon and 115 in the A horizon. In forest stands with shrub participation, the number of fungal genera was higher, reaching 236, 244, and 267 in the O horizon and 164, 165, and 186 in the A horizon for stands with rowan, alder buckthorn, and hazel, respectively. Similarly, for bacterial genera, pine monocultures had the lowest numbers (102 genera in the O horizon and 105 in the A horizon). In stands with the presence of shrubs, the total number of bacterial genera increased to 157, 170, and 225 in the O horizon, and to 143, 132, and 160 in the A horizon, respectively, for stands with rowan, alder buckthorn, and hazel (Fig. 1).

**Fig. 2:**
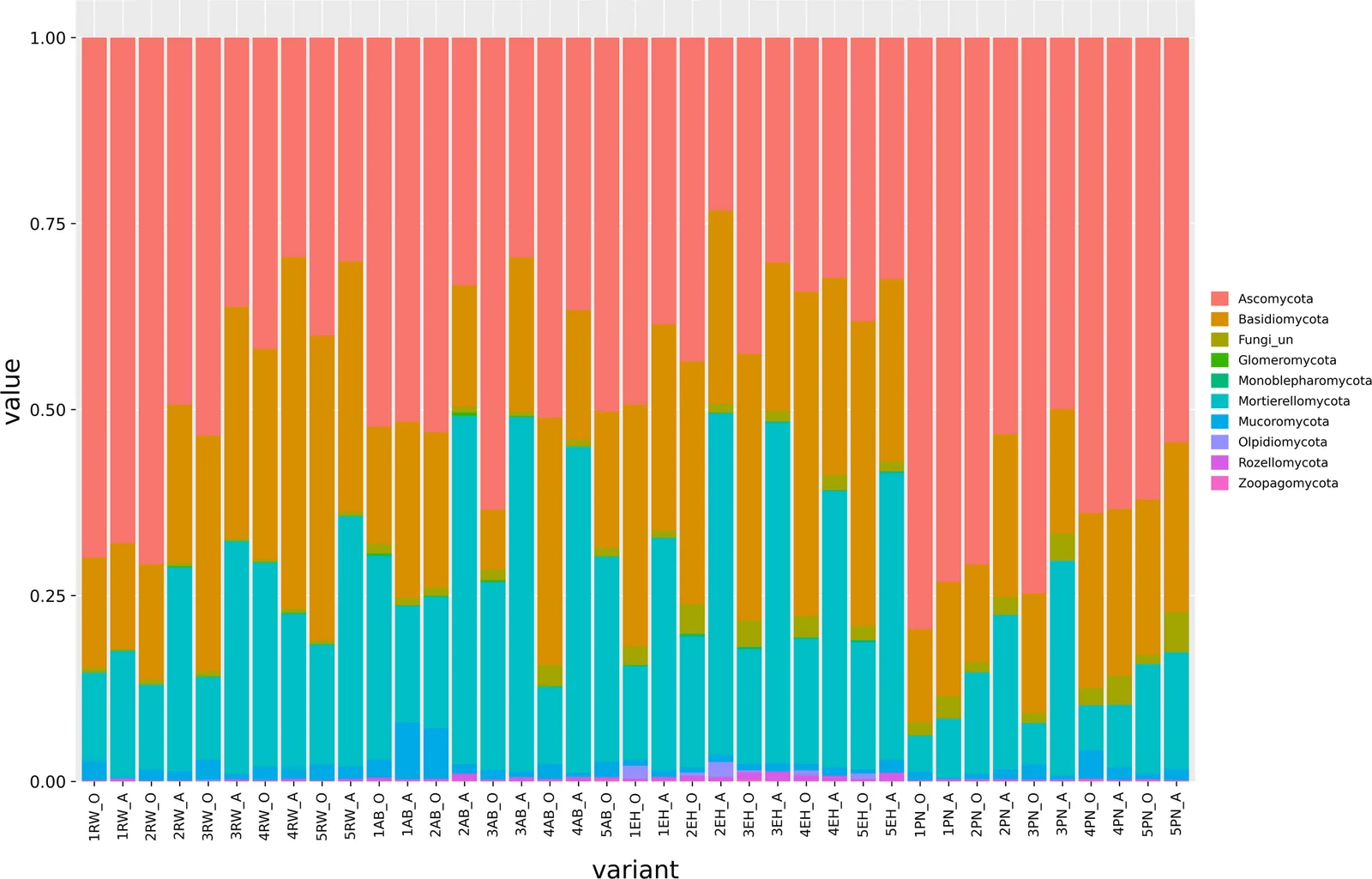
Relative abundance of fungal phyla identified in soil under influence of different species (AB – alder buckthorn, EH – European hazelnut, RW – rowan, PN – pine; O – organic horizon, A – humus mineral horizon)

**Fig. 3:**
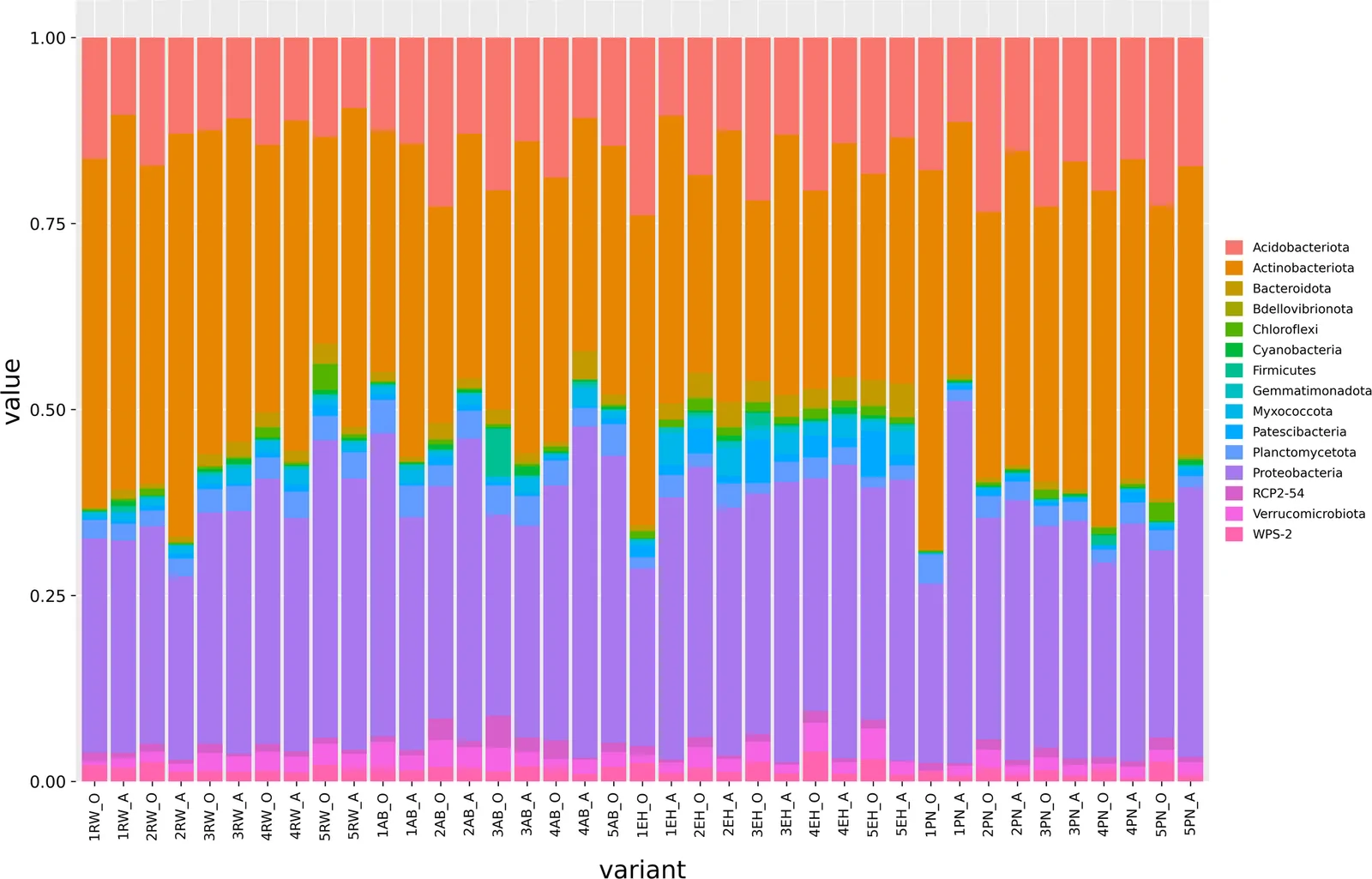
Relative abundance of bacterial phyla identified in soil under influence of different species (AB – alder buckthorn, EH – European hazelnut, RW – rowan, PN – pine; O – organic horizon, A – humus mineral horizon)

The dominant fungal phyla across all soils were Ascomycota, Basidiomycota, and Mortierellomycota (Fig. 2). Ascomycota clearly dominated in pine monocultures (70% and 59% relative abundance in the O and A horizons, respectively), whereas their proportion decreased in stands with shrubs. In contrast, Mortierellomycota were consistently more abundant in shrub stands than in pine monocultures in both soil horizons. Basidiomycota were relatively more abundant under rowan and European hazelnut, while their abundance under alder buckthorn was similar to that in pine stands. Other fungal phyla occurred at low relative abundances (< 3%). Among bacteria, the most abundant phyla were Actinobacteriota, Proteobacteria, and Acidobacteriota (Fig. 3). The first of these groups showed a relative abundance of 35–47% in organic horizons and tended to increase in stands with rowan understory. In the humus-humus mineral horizons, Actinobacteriota accounted for 29–41%, with lower values in stands with European hazelnut and alder buckthorn, and higher values in stands with rowan and in pine monocultures. The Proteobacteria phylum accounted for 31–37% of the organic horizons, with the lowest share observed under the rowan understory. In humus mineral horizons, their abundance ranged from 27 to 33%, with a tendency to increase in the presence of all shrub species. The Acidobacteriota phylum accounted for 11–15% in organic horizons and 15–21% in humus mineral horizons, with the lowest values observed under the rowan understory. Other bacterial phyla were less abundant. Planctomycetota accounted for 1.8–3.7% (with no significant differences between variants), while Verrucomicrobiota showed the highest abundance in humus mineral horizons under European hazelnut and alder buckthorn (2.9%). The phyla *WPS-2* and *Patescibacteria* reached their highest relative abundances in humus mineral horizons under European hazelnut (2.8% and 3.9%, respectively), and *RCP2-54* was most abundant in humus mineral horizons under alder buckthorn (2.6%) (Fig. 3).

**Fig. 4:**
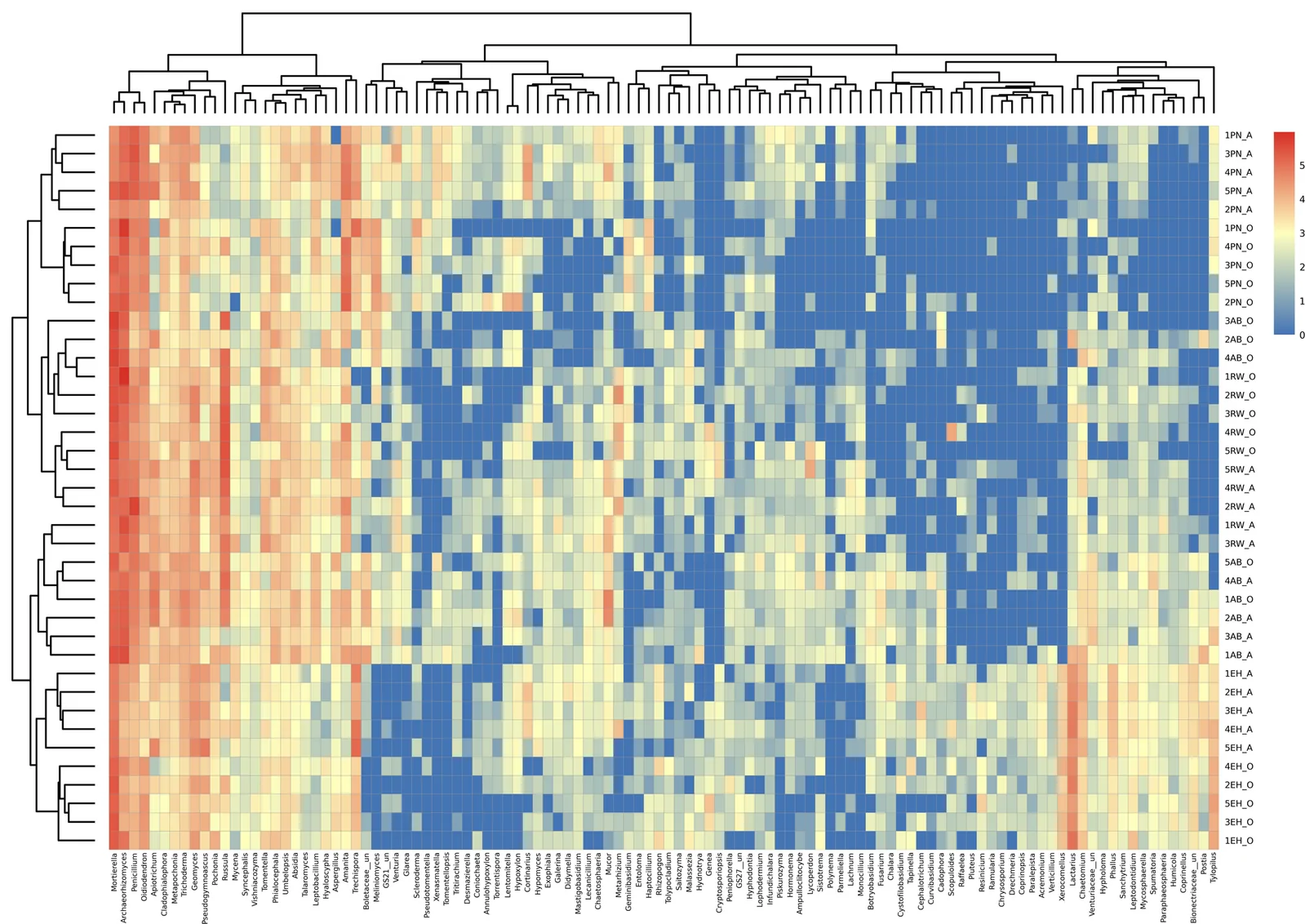
Abundance heatmap based on the Illumina metabarcoding data constructed using log10 + 1-transformed abundance data for fungal species whose total number of reads exceeded 400 (AB – alder buckthorn, EH – European hazelnut, RW – rowan, PN – pine; O – organic horizon, A – humus mineral horizon)

**Fig. 5:**
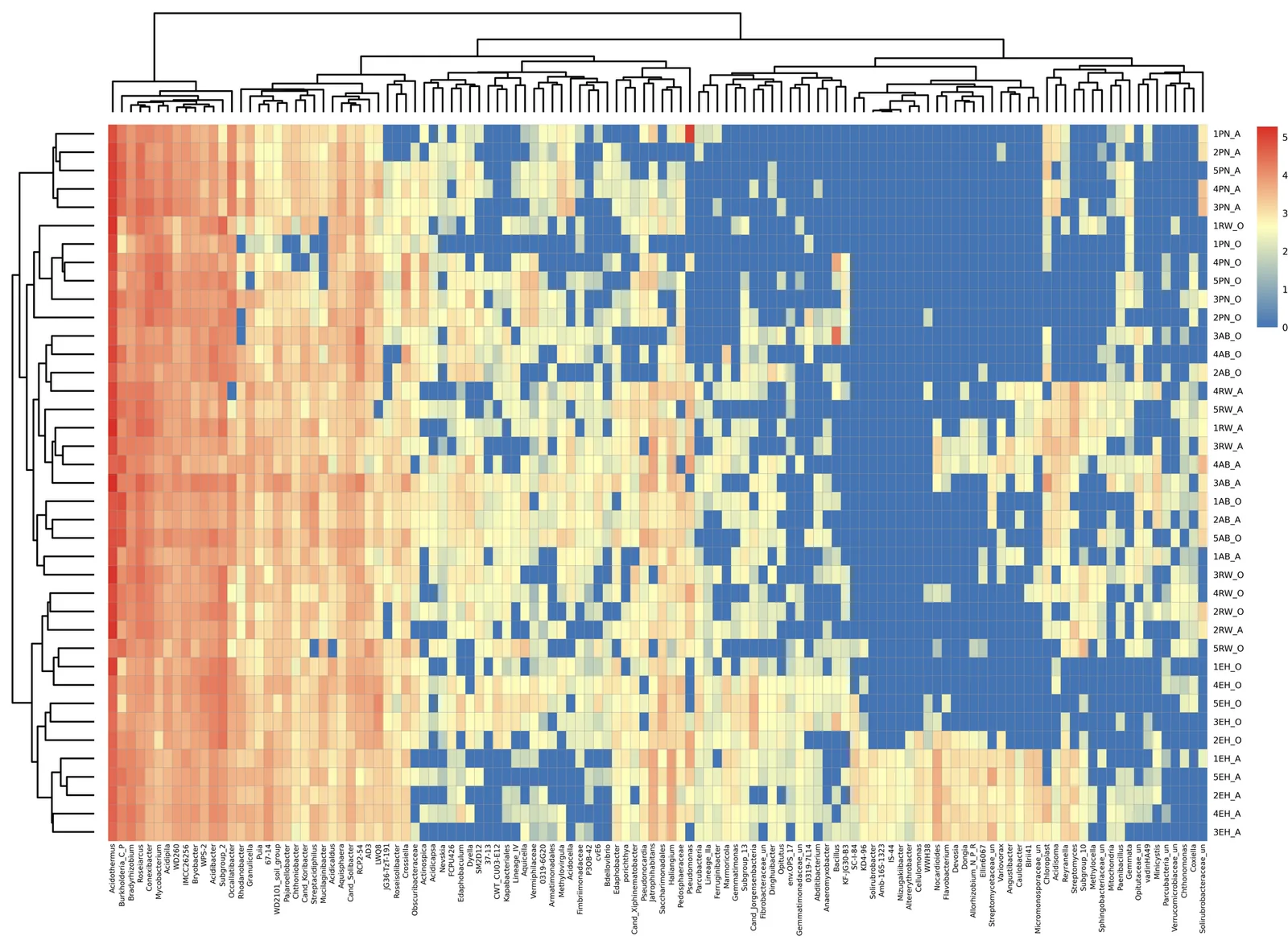
Abundance heatmap based on the Illumina metabarcoding data constructed using log10 + 1-transformed abundance data for bacterial species whose total number of reads exceeded 400 (AB– alder buckthorn, EH – European hazelnut, RW – rowan, PN - pine; O – organic horizon, A – humus mineral horizon)

The analysis of fungal genera in the studied forest stands revealed distinct differences in community structure among shrub species (Figs. 4 and 5). In the soil of pine stands without shrub understory, the most abundant fungal genus was *Archaeorhizomyces* (25.5% relative abundance), followed by *Penicillium* (13.4%), *Mortierella* (12.6%), *Amanita* (9.4%), *Oidiodendron* (7.8%), and an unidentified genus of *Helotiales* fungi (4.4%). In stands with rowan understory, in addition to the dominance of *Archaeorhizomyces* and *Mortierella* (both 20.8% relative abundance), the most abundant genera included *Russula* (16.8%) and *Penicillium* (10.1%). In stands with alder buckthorn, the dominant fungal genera were *Mortierella* and *Archaeorhizomyces* (29.1% and 19.6%, respectively), followed by *Russula*, *Penicillium*, and *Apiotrichum* (7.2%, 5.0%, and 3.8%, respectively). In soils under stands with European hazelnut understory, besides the dominance of *Mortierella* (27.7%), the most abundant genera included *Lactarius* (10.3%), *Trechispora* (6.3%), *Archaeorhizomyces* (5.0%), *Geomyces* (4.3%), and *Pseudogymnoascus* (3.4%). A relatively large number of genera occurred in low abundance (< 1%) in the studied soils. Among those that distinguished stands with European hazelnut understory (showing significantly higher relative abundances compared to other stand types) were *Chaetomium*, *Xerocomellus*, *Phallus*, *Gymnopus*, *Isaria*, and *Leptodontidium* (Fig. 4). In the case of bacteria, a much greater diversity of genera was observed, with *Acidothermus* being the most abundant genus across all studied stand types, ranging from 12.1% in stands with European hazelnut understory to 24.9% in those with rowan understory (Fig. 5). In addition to this genus, an unidentified genus of *Acidimicrobiia* bacteria showed relatively high abundance, ranging from 4.3% in soils under European hazelnut understory to 6.8% under alder buckthorn. The genus *Burkholderia–Paraburkholderia* was also relatively abundant, reaching its highest value (7.2%) in soils under alder buckthorn, whereas in other forest types its share ranged from 2.8% to 4.1%. A similar relative abundance (3–5%) was found for *Roseiarcus* and unidentified bacterial genera belonging to *Elsterales*, *Acidobacteriales*, and *Xanthobacteraceae*. In stands with rowan and European hazelnut understory, higher relative abundances were recorded for *Bradyrhizobium* and *Subgroup 2* bacteria (3.5–4%), whereas in pine monocultures and stands with alder buckthorn their proportions were significantly lower (1.4–2.2%). In the presence of European hazelnut understory, a higher relative abundance of *Acidibacter* was also observed. Among bacterial genera with moderate abundance (1–2%), three were significantly more numerous in pine monocultures: *Acidipila*, *Mycobacterium*, and *Occallatibacter*. Other genera, such as *IMCC26256*, *Bryobacter*, *WD260*, and *RCP2-54*, showed similar abundances across all stand types.

**Fig. 6:**
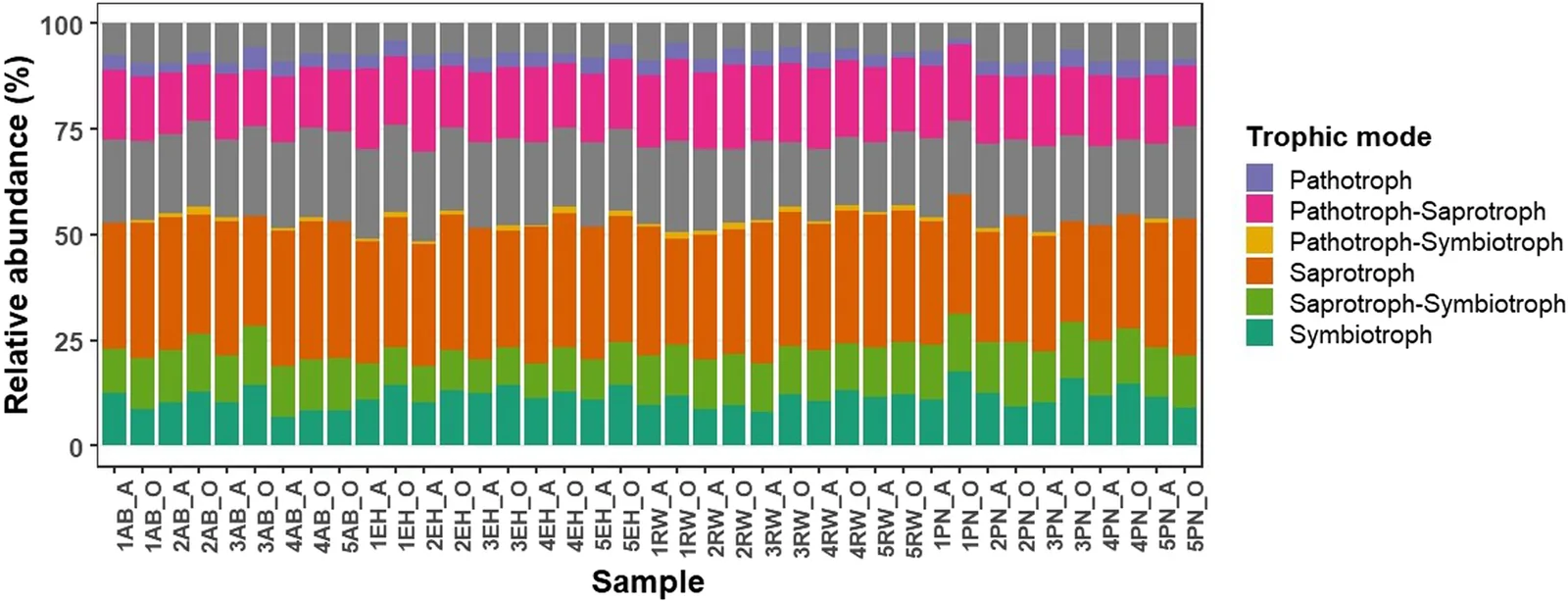
Functional structure of fungal communities based on FUNGuild trophic mode classification across shrub species and soil horizons

In soils under European hazelnut understory, among the less frequent genera, several showed significantly higher relative abundance than in pine monocultures, including *Solibacter*, *6114*, *LWQ8*, *Rhodanobacter*, *Jatrophihabitans*, *Mucilaginibacter*, and *Haliangium*. In stands with alder buckthorn, higher relative abundances were found for the *WD2101 Soil group*, *Streptacidiphilus*, and *Candidatus Koribacter* (Fig. 5). Fungal communities across all analysed samples were dominated by saprotrophic fungi, which accounted for the largest proportion of the identified trophic modes (Fig. 6). Considerable contributions were also observed in mixed trophic groups, particularly in pathotroph–saprotroph and saprotroph–symbiotroph fungi, indicating the multifunctional ecological roles of many taxa in the studied soils. Symbiotrophic fungi occurred at moderate abundances across all stand types, whereas strictly pathogenic fungi represented only a small fraction of the communities. Differences among stand variants were mainly associated with shifts in the relative abundance of saprotrophic and symbiotrophic groups. In particular, soils under shrub admixtures tended to show higher proportions of symbiotrophic and saprotroph–symbiotroph fungi than pine monocultures, suggesting that the presence of understory shrubs promotes greater functional complexity in soil fungal communities (Fig. 6).

### Relationships between soil properties and microbial communities

**Fig. 7:**
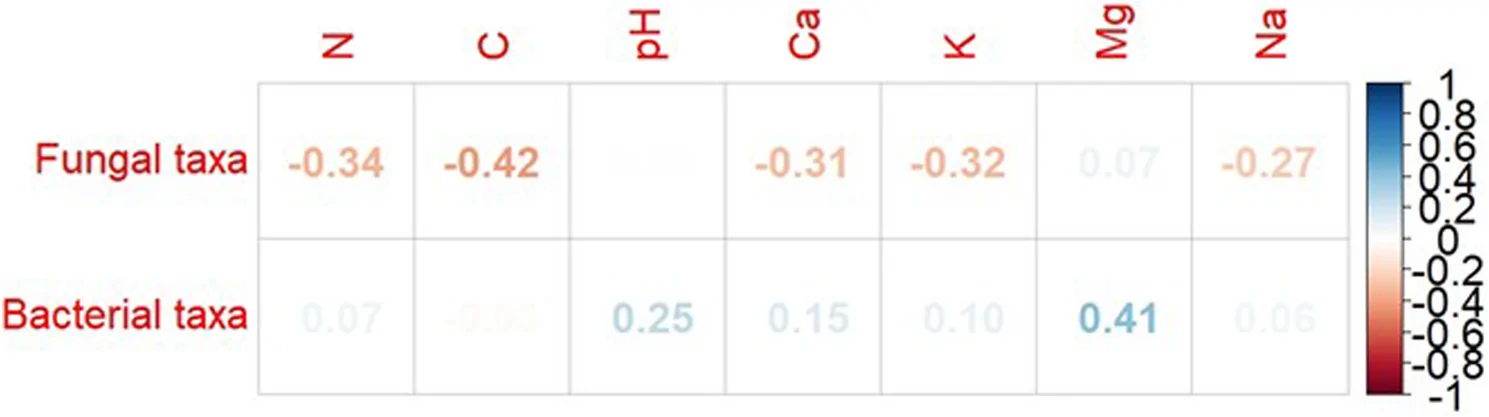
Spearman correlations between fungal, bacterial taxa and properties of the studied soils

**Fig. 8:**
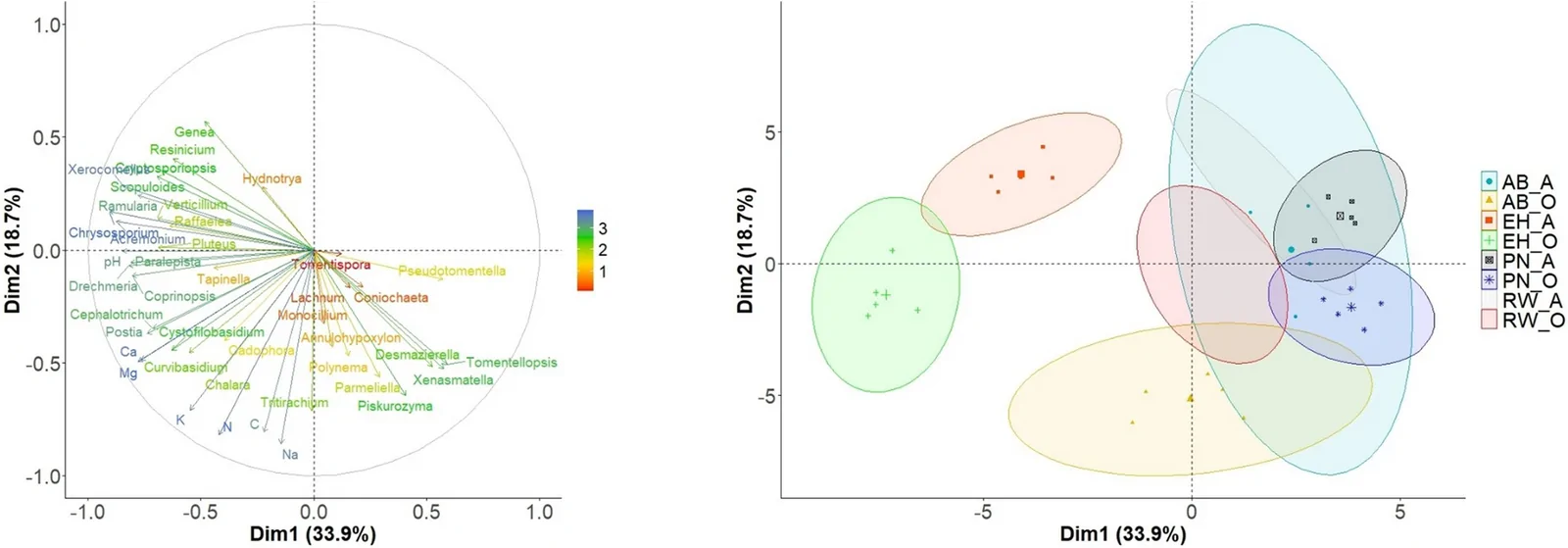
PCA diagram with the projection of variables of soil properties and fungal taxa on the plane of the first and second factors

**Fig. 9:**
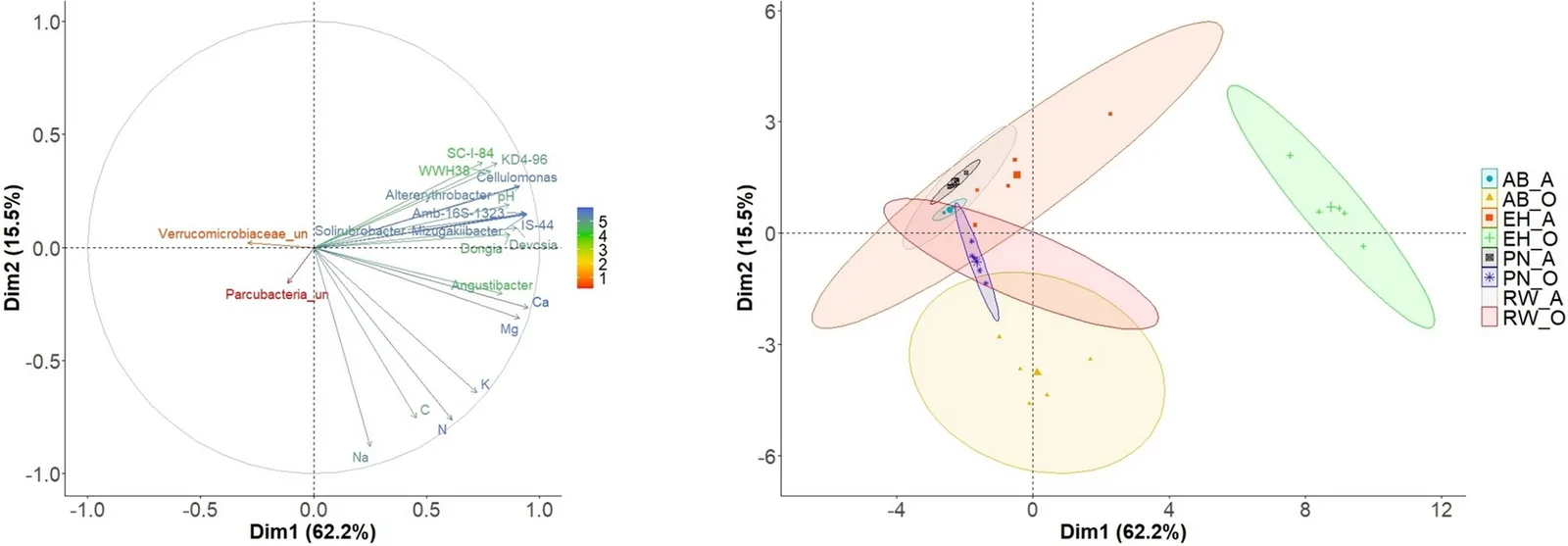
PCA diagram with the projection of variables of soil properties and bacterial taxa on the plane of the first and second factors

An additional analysis was performed to assess the relationships between identified microbial groups and selected soil properties determined in the humus accumulation horizons of the studied stands (Fig. 7). A selection of bacterial genera was made, comprising taxa with the strongest associations with specific soil parameters. The PCA results indicate that the variability in fungal community composition is largely associated with soil chemical parameters (Fig. 8). The first two principal components together explain 52.6% of the total variance (Dim1–33.9%, Dim2–18.7%). The PCA ordination showed that vectors representing soil pH and exchangeable base cations (Ca, Mg, and K) were closely grouped and oriented in a similar direction, indicating strong positive correlations among these variables and their joint influence on fungal community structure. Fungal genera located near these vectors, including Tomentella, Pseudotomentella, Desmazierella, Xenasmatella, and Lachnum, were positively associated with soils characterised by higher pH and increased concentrations of base cations. In contrast, vectors representing organic carbon and nitrogen contents formed a separate gradient associated with genera such as Curvibasidium, Cephalotrichum, Chalara, and Coprinopsis. Overall, the PCA analysis indicates that soil acidity and nutrient availability were the main factors differentiating fungal communities among the analysed stands. The PCA analysis also revealed clear differences in the structure of fungal communities among forest stand types and soil horizons. Samples from the organic and humus mineral horizons formed distinct clusters, confirming a significant effect of soil horizon on the microbial composition. The most pronounced differentiation was observed between soil samples under European hazelnut, rowan, and pine. Soils from stands with shrub participation formed separate groupings from the pine monoculture, indicating that the presence of shrubs promotes the development of distinct fungal communities, likely due to greater diversity of organic substrates and increased availability of mineral nutrients (Fig. 8). The first two principal components (Dim1 and Dim2) together explain 77.7% of the total variance in the data (Dim1–62.2%, Dim2–15.5%), indicating a strong relationship between the structure of bacterial communities and the analysed soil chemical properties (Fig. 9). The factors that had the greatest influence on microbial composition differentiation were nitrogen (N), carbon (C), calcium (Ca), magnesium (Mg), and soil pH. Directional relationships indicate that certain bacterial taxa, including *Cellulomonas*, *Dongia*, *Devosia*, *Mizugakiibacter*, *Solirubacter*, *Angustibacter*, and *SC-I-84*, exhibit a positive correlation with higher pH values and increased concentrations of base cations. In contrast, the families *Verrucomicrobiaceae_un* and *Parcubacteria_un* were associated with soils of lower mineral nutrient content, characterised by lower pH values and cation concentrations. The analysis of sample distribution revealed clear differences in bacterial community structure among forest stand types and soil horizons. Samples from the organic and humus mineral horizons formed distinct clusters, confirming a significant effect of soil horizon on bacterial composition. The most pronounced differentiation was observed between soil samples collected under European hazelnut (EH**)**, rowan (RW), and pine (PN). Soils from stands with shrub participation (EH, RW, AB) formed distinct groups compared with the pine monoculture, indicating that shrubs influence the development of distinct bacterial communities (Fig. 9).

Alpha diversity indices revealed clear differences in the structure of bacterial and fungal communities among the analysed stand variants and soil horizons (Table 3). In general, bacterial communities showed higher Shannon diversity and Pielou’s evenness than fungal communities. The highest bacterial diversity was observed in soils under European hazelnut understory, particularly in the organic horizon, whereas lower values were recorded in pine monocultures and stands with rowan understory. Fungal communities showed lower evenness and greater dominance than bacterial communities, as indicated by lower Pielou and Simpson index values. The highest fungal diversity was also observed in soils under European hazelnut understory, whereas the lowest values were observed in pine monocultures. In both microbial groups, diversity indices tended to be higher in the organic horizons than in the humus mineral horizons (Table 3).

**Table 3:** Alpha diversity indices (Shannon diversity, Simpson diversity, and Pielou evenness) of bacterial and fungal communities in organic (Ofh) and humus mineral (A) soil horizons under different shrub understory variants in Scots pine stands (mean ± SD)

Community	Stand variant	Soil horizon	Shannon index (H)	Simpson index (D)	Pielou evenness (J)
Bacteria	AB	Ofh	4.60 ± 0.16	0.98 ± 0.01	0.82 ± 0.02
		A	4.51 ± 0.16	0.98 ± 0.01	0.83 ± 0.01
	EH	Ofh	5.01 ± 0.16	0.99 ± 0.01	0.87 ± 0.01
		A	4.66 ± 0.32	0.98 ± 0.01	0.84 ± 0.04
	RW	Ofh	4.21 ± 0.16	0.95 ± 0.01	0.77 ± 0.03
		A	4.29 ± 0.35	0.96 ± 0.02	0.79 ± 0.05
	PN	Ofh	4.00 ± 0.35	0.96 ± 0.02	0.78 ± 0.03
		A	4.17 ± 0.35	0.97 ± 0.02	0.80 ± 0.02
Fungi	AB	Ofh	3.52 ± 0.35	0.92 ± 0.04	0.62 ± 0.05
		A	2.77 ± 0.35	0.85 ± 0.06	0.52 ± 0.07
	EH	Ofh	3.85 ± 0.35	0.95 ± 0.02	0.68 ± 0.05
		A	3.40 ± 0.35	0.93 ± 0.01	0.63 ± 0.03
	RW	Ofh	3.29 ± 0.35	0.90 ± 0.04	0.58 ± 0.03
		A	2.85 ± 0.35	0.86 ± 0.10	0.53 ± 0.08
	PN	Ofh	3.32 ± 0.35	0.93 ± 0.02	0.61 ± 0.04
		A	2.69 ± 0.35	0.85 ± 0.06	0.54 ± 0.05

*AB* alder buckthorn, *EH* European hazelnut, *RW* rowan, *PN* pine

The GLM analysis showed that species identity significantly affected the Shannon diversity index (F = 3.58, *p* = 0.0178), whereas its effect on the Simpson diversity index (*p* = 0.0860) and Pielou evenness (*p* = 0.1661) was not significant (Table 4). Soil horizon also significantly influenced Shannon diversity (F = 3.72, *p* = 0.0479) and Simpson diversity (F = 4.02, *p* = 0.0488), while no significant effect was detected for Pielou evenness (*p* = 0.2808). The interaction between species and soil horizon was not significant for any of the analysed diversity indices (*p* > 0.05), indicating that the effects of shrub species on microbial diversity were consistent across soil horizons (Table 4).

**Table 4:** Summary of the GLM analysis for the diversity indices (Shannon diversity, Simpson diversity, and Pielou evenness)

	Shannon index (H)		Simpson index (D)		Pielou evenness (J)	
	F	*p*	F	*p*	F	*P*
Species (S)	3.58	0.0178	2.29	0.0860	1.74	1.1661
Horizon (H)	3.72	0.0479	4.02	0.0488	1.18	0.2808
S*H	0.14	0.9348	0.16	0.9232	0.07	0.9770

A statistically significant effect is marked in red

**Fig. 10:**
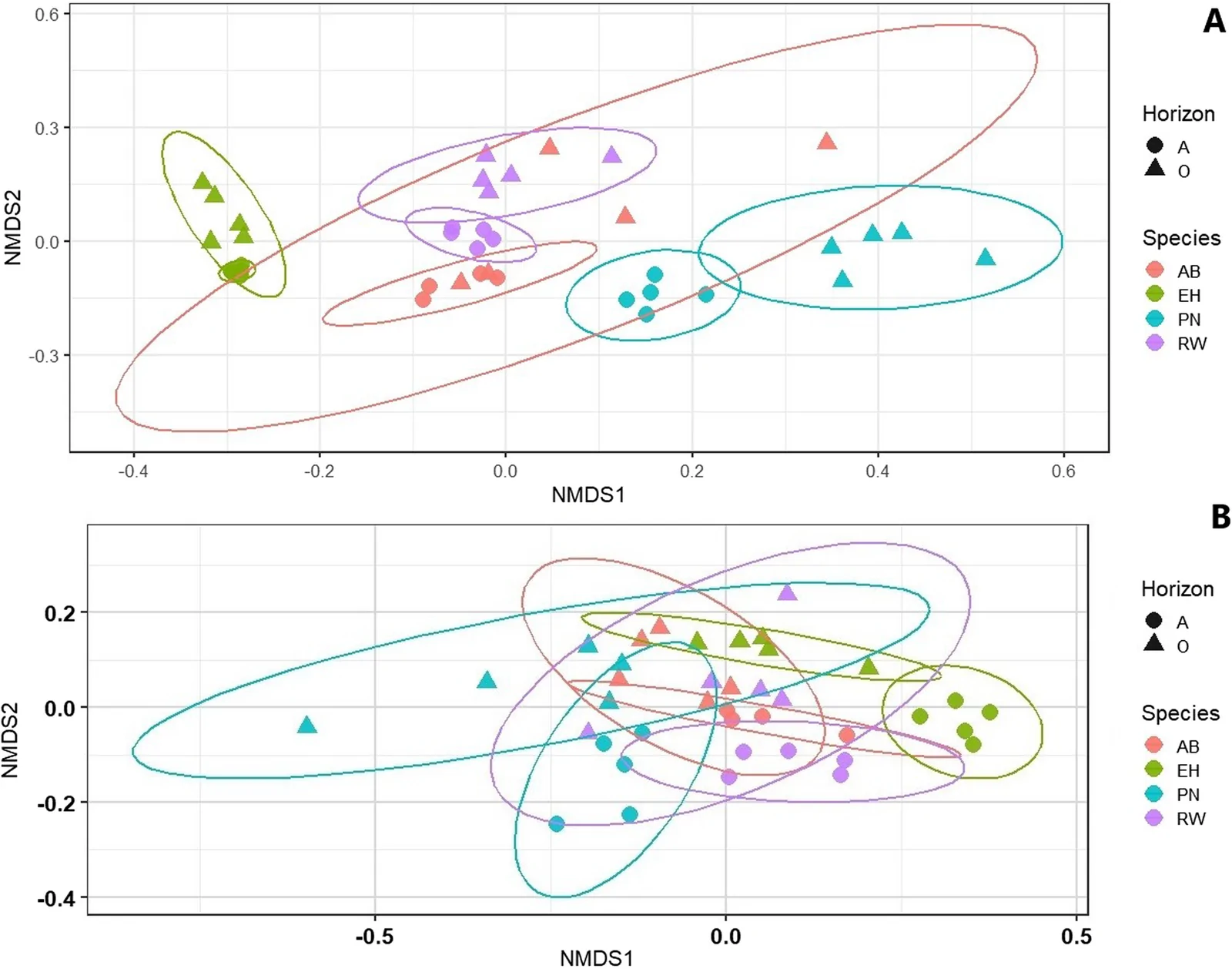
NMDS ordination of fungal (**A**) and bacterial (**B**) community composition based on Bray – Curtis dissimilarities

Pairwise PERMANOVA analyses revealed significant differences in both bacterial and fungal community composition among all shrub species (*p* < 0.01 for all comparisons) (Table 5). For bacterial communities, the strongest separation was observed between EH and PN (R² = 0.430, pseudo-F = 13.58), whereas the weakest difference occurred between AB and RW (R² = 0.206, pseudo-F = 4.41). Similar patterns were found for fungal communities, with the largest divergence detected between EH and PN (R² = 0.618, pseudo-F = 29.14). These results indicate that the identity of understory shrub species strongly influenced soil microbial community composition (Table 5). The NMDS ordination analyses also demonstrated a clear separation of samples by shrub species and soil horizons, indicating that both understory vegetation and soil horizons strongly influenced the composition of soil microbial communities (Fig. 10). Distinct clustering patterns were particularly evident for stands with European hazelnut and alder buckthorn understories, suggesting species-specific effects on belowground microbial assemblages.

**Table 5:** Pairwise PERMANOVA comparisons of bacterial and fungal community composition among understory shrub species based on Bray–Curtis dissimilarities

	Comparison	pseudo-F	R²	p
Bacteria	AB vs. EH	7.21	0.298	0.002
	AB vs. PN	6.23	0.268	0.002
	AB vs. RW	4.41	0.206	0.002
	EH vs. PN	13.58	0.430	0.002
	EH vs. RW	6.05	0.252	0.002
	PN vs. RW	7.58	0.296	0.002
Fungi	AB vs. EH	12.54	0.424	0.002
	AB vs. PN	7.65	0.310	0.002
	AB vs. RW	5.21	0.234	0.002
	EH vs. PN	29.14	0.618	0.002
	EH vs. RW	16.88	0.484	0.002
	PN vs. RW	12.38	0.407	0.002

*AB* alder buckthorn, *EH* European hazelnut, *RW* rowan, *PN* pine; statistical significance was accepted at *p* < 0.05

## Discussion

The conducted analyses confirmed the first research hypothesis that the presence of shrubs in temperate pine forests alters the composition of soil microbial communities. In the conducted study, we observed a significant increase in the diversity of both fungal and bacterial genera in the soils of pine stands with an understory of all the studied shrubs (rowan, alder buckthorn, and European hazelnut) compared to pine monocultures. In the case of fungi, the increase ranged from 30 to 47% in the organic horizons and 42–62% in the humus mineral horizons. For bacteria, the number of detected genera increased by 54–121% in the organic horizons and by 26–52% in the humus mineral horizons. The most significant increase in microbial diversity was consistently observed in the presence of a European hazelnut understory. The positive effect of admixture species on soil chemical and biological properties in conifer monocultures may be explained by several mechanisms described in previous studies [38–42]. One of the most important is the enrichment of soil organic matter resulting from a more diverse litter input. Admixture species can take up nutrients from deeper soil horizons, accumulate them in aboveground biomass, and subsequently return them to the soil through litterfall, thereby enhancing nutrient cycling and improving soil fertility [38–42]. The studied shrub species have higher nutrient demands than pine (per unit of biomass), which indicates a stronger nutrient uptake from deeper soil horizons and their incorporation into aboveground organs (leaves, twigs, fruits, etc.). Plant litter accumulating on the soil surface serves as a major source of nutrients that, following decomposition and humification, enrich the upper organic and mineral soil horizons.

The present study confirmed both the second and third hypotheses. Shrub-induced differences in soil chemical properties, particularly higher pH and nutrient availability, were associated with differences in microbial community composition and diversity. These factors may represent important mechanisms underlying the observed patterns, although they were not directly tested in this study. Moreover, these effects were most pronounced in the surface organic horizons, where litter inputs, root exudates, and rhizosphere activity are concentrated. Stokdyk and Herrman [43] demonstrated that freshly fallen leaves of alder buckthorn contained significantly higher nitrogen concentrations compared to material derived from *Pinus resinosa*, *P. strobus*, *Acer rubrum*, *Betula papyrifera*, *Populus tremuloides*, and *Prunus serotina*. The more diverse litter input associated with understory shrubs may partly explain the observed increase in microbial diversity by providing a broader range of organic substrates for soil microorganisms [44]. However, litter quality and decomposition dynamics were not directly assessed in this study. The chemical composition of humus horizons under the studied stands (both monocultures and mixed stands with shrub species) indicates a significant enrichment in exchangeable base cations, a decrease in acidity, and an increase in soil pH. This confirms the presence of a specific “nutrient pump” in understory stands, with the most substantial effect observed in the presence of European hazelnut [13]. A second mechanism potentially involved in shrub-induced changes in soil properties may be associated with the activity and development of their root systems, consistent with previous reports. Their intensive growth and branching increase belowground biomass and root exudation, promoting greater biological diversity in rhizosphere zones. Studies of the rhizosphere of the examined shrub species indicate greater exudate release, resulting in a significant increase in soil biochemical activity [10, 13]. Enhanced biochemical activity in the root zone has been reported to increase nutrient availability and uptake in previous studies [45, 46]. In the present study, these processes may have contributed to the observed differences in microbial communities but were not directly quantified. Fine roots are a crucial component of forest ecosystems, contributing to nutrient cycling rates, and their decomposition is strongly linked to soil N and P availability, as well as mean annual temperature and precipitation [44]. It has also been shown that the presence of shrubs and the development of a more complex vertical forest structure modify microclimatic conditions (by shading the forest floor and increasing humidity), thereby creating diverse microhabitats favourable to higher biodiversity [45, 46]. Together, improved litter quality, intensified rhizosphere processes, enhanced nutrient availability, and moderated microclimatic conditions represent plausible mechanisms that may explain the higher microbial diversity observed in stands with shrub understories. However, these mechanisms were inferred from previous studies and were not directly examined here.

Several interrelated factors may explain the particularly favourable effect of the European hazelnut understory on microbial diversity in pine stands. Field observations suggest that European hazelnut tends to form a relatively well-developed understory layer with comparatively high aboveground biomass, likely accompanied by increased belowground biomass. Field measurements confirmed that the European hazelnut has the most extensive root system, characterised by the highest root biomass, total root length, and the largest number of fine roots and root tips [10]. Root exudate analyses also showed that European hazelnut roots release the highest amounts of organic carbon, resulting in a marked enrichment of the humus horizons and stimulation of soil biochemical activity. Additionally, humus accumulation horizons under European hazelnut were found to have the most favourable chemical properties, such as the highest concentrations of exchangeable cations, phosphorus, and pH [10, 13], confirming the strongest “nutrient pump” effect in the presence of this species. Consequently, the European hazelnut understory was associated with the highest microbial diversity, and this pattern may be related to greater organic matter inputs, more favourable soil chemical properties, and more extensive root systems documented in previous studies [10, 13].

The FUNGuild analysis indicated that saprotrophic fungi constituted the dominant functional group in all analysed soils, highlighting the major role of decomposition processes in the studied forest ecosystems. The substantial proportion of mixed trophic groups, particularly pathotroph–saprotroph and saprotroph–symbiotroph fungi, suggests high functional plasticity in soil fungal communities and the coexistence of microorganisms capable of exploiting multiple ecological strategies in response to environmental conditions. The increased proportion of symbiotrophic and saprotroph–symbiotroph fungi in soils under shrub admixtures may reflect the greater diversity of root-associated niches and enhanced availability of organic substrates in these stands. Shrub species likely contribute to the development of more functionally complex fungal communities by increasing litter inputs, intensifying rhizosphere processes, and improving soil chemical conditions. Similar relationships between shrub presence, plant functional traits, and shifts in fungal functional structure have been reported in previous studies examining shrub-driven changes in soil microbial communities [26]. The relatively low abundance of strictly pathogenic fungi may additionally indicate that soils under shrub admixtures provide more stable and biologically balanced conditions, favouring mutualistic and decomposer fungi over pathogenic taxa.

Another factor contributing to the increase in microbial diversity in stands with understory shrubs is the emergence of new host species. Each woody species is known to associate with a specific set of microorganisms forming mycorrhizal symbioses, and these sets may differ among hosts [47]. However, there is evidence that the composition of root-colonising microflora also depends on the physiological condition of the host and the environmental conditions in which its aboveground parts develop [48]. The presence of new types of organic matter creates “micro-niches,” providing habitats for microorganisms with specific enzymatic abilities. In this way, understory shrubs increase the structural and biochemical complexity of the soil environment, enhancing habitat heterogeneity and facilitating the establishment of specialised microbial taxa occupying distinct ecological niches.

The increase in biodiversity also extends to other groups of organisms. Stiegel et al. [49] showed that the presence of shrubs in forest stands enhances insect diversity, particularly among Diptera, Hemiptera, and Thysanoptera. In dense shrub layers, allelopathic interactions may additionally influence the formation of microbial communities. The accumulation of specific decomposition products of organic matter (such as alkaloids, terpenes, and other biologically active plant compounds) may affect microbial assemblages inhabiting the rhizosphere and humus horizons or even directly influence the roots of other tree species [50].

Although there is no direct evidence of strong allelopathic effects from the studied shrub species, it cannot be ruled out that the decomposition products of their organic matter possess allelochemical properties influencing other species, and that the observed microbial structure results partly from such interactions. Among the studied shrubs, alder buckthorn is particularly rich in biologically active compounds, including glycosides (frangulin derivatives), flavonoids (including rhamnetin), tannins, and peptide alkaloids, all of which have long been recognised for their medicinal properties [51]. For example, anthraquinone glycosides from alder buckthorn (*glucofrangulin*, *frangulin*) exhibit antimicrobial activity [52, 53]. Stokdyk and Herrman [43] studied the effects of *Frangula alnus* on soil microbial structure and selected biochemical properties (in their study area, this species was non-native). The presence of this shrub in mixed stands (with *Acer rubrum*, *Betula papyrifera*, *Pinus strobus*, *Pinus resinosa*, *Populus tremuloides*, *Prunus serotina*, and *Quercus* spp.) resulted in specific positive changes, including higher nitrogen mineralisation rates and a tendency toward increased microbial biomass carbon (MBC) in the soil.

Although shrub species significantly affected the Shannon diversity index, their influence on Simpson diversity and Pielou evenness was less pronounced. This suggests that understory shrubs primarily altered the composition and relative abundance of microbial taxa rather than causing substantial changes in overall community evenness. Such a pattern is consistent with the PERMANOVA results, which revealed clear differences in community composition among shrub species. Together, these findings indicate that shrub species identity mainly promotes species turnover rather than uniformly increasing or decreasing microbial diversity. The significant effect of soil horizon on Shannon and Simpson diversity further emphasises that vertical heterogeneity of the soil profile remains an important determinant of microbial diversity. Differences in organic matter accumulation, substrate availability, and microenvironmental conditions between the organic and mineral horizons likely create distinct ecological niches for microorganisms [3, 41]. The lack of a significant shrub species × soil horizon interaction suggests that the response of microbial diversity to shrub identity was generally consistent across the two soil horizons.The functional guild analysis suggests that shrub admixture may influence not only the taxonomic composition of fungal communities but also their ecological functions. The predominance of saprotrophic fungi indicates a high decomposition potential, which may accelerate litter breakdown and nutrient mineralisation, thereby increasing nutrient availability for plants [54]. The relatively greater proportion of symbiotrophic and saprotroph–symbiotroph fungi in soils under shrub species may further enhance nutrient acquisition through mycorrhizal associations and contribute to greater stability of soil ecosystem processes [55]. Although differences among shrub species in functional guild composition were generally moderate, European hazelnut tended to support a higher proportion of fungi associated with symbiotic functions, consistent with its stronger positive effects on soil properties and overall microbial diversity. The consistently low proportion of pathotrophic fungi across all treatments may also indicate that shrub admixtures do not promote fungal pathogens but rather favour decomposer communities.

Although the study demonstrated clear relationships among understory shrubs, soil properties, and microbial community composition, some ecological mechanisms discussed, including root exudation intensity and belowground biomass allocation, were not directly measured and should therefore be interpreted as potential processes supported by previous studies. In addition, the study was conducted within a specific forest type and environmental setting, which may limit the broader generalisation of the results. Future studies combining microbial analyses with direct measurements of rhizosphere processes, root traits, and microclimatic conditions across different forest ecosystems would provide a more comprehensive understanding of shrub–soil–microbiome interactions.

## Conclusions

Soil chemical properties and microbial community composition differed significantly among the analysed stand types and soil horizons. The presence of understory shrubs, particularly European hazelnut and alder buckthorn, was associated with higher nutrient availability, reduced soil acidity, and improved overall soil chemical status compared with pine monocultures. These conditions promoted greater fungal and bacterial diversity, especially within organic horizons, with the highest microbial richness recorded in stands containing European hazelnut. Shrub admixtures also altered the taxonomic structure of soil microbiota, increasing the relative abundance of Basidiomycota and Mortierellomycota. Overall, the results indicate that incorporating shrub species into Scots pine stands may enhance soil function, stimulate nutrient cycling, and contribute to the development of more biologically diverse and ecologically resilient forest ecosystems.

## Supplementary Information

Supplementary Material 1.

## Data Availability

Libraries with the fungal sequences analysed have been deposited with the GenBank (https://www.ncbi.nlm.nih.gov/) under project number PRJNA1357095, and those with the bacterial sequence have been deposited under project number PRJNA1357109.
