# Supplementary material for: Species-specific effects of understory shrubs on soil chemistry and microbial communities in temperate Scots pine forests
Source: BMC Plant Biol. 2026 Jul 14;26:1726. doi: 10.1186/s12870-026-09514-6 (PMC13647620; doi:10.1186/s12870-026-09514-6)
Supplement: Supplementary file 1 [file 12870_2026_9514_MOESM1_ESM.docx]

Table S1. Geographic coordinates of all sampling sites across the studied forest districts in Poland.Supplementary information.

Adam Górski, Jarosław Lasota, Marta Kempf*, Ewa Błońska

Department of Ecology and Silviculture, Faculty of Forestry, University of Agriculture, 29 Listopada 46 Str., 31-425 Kraków, Poland

* Corresponding author: m.kempf@ur.krakow.pl

Table S1.

| Forest District | Latitude  N | Longitude  E |
| --- | --- | --- |
| Olkusz | 50.2813 | 19.5650 |
| Koniecpol | 50.7760 | 19.6880 |
| Włoszczowa | 50.8526 | 19.9659 |
| Przedbórz | 51.0850 | 19.8733 |
| Smardzewice | 51.5276 | 20.0410 |
